# Access pathways to the transsexualizing process in Brazil: a scoping review

**DOI:** 10.1590/S2237-96222024v33e2024311.especial.en

**Published:** 2025-01-10

**Authors:** Mariluza Sott Bender, Ingre Paz, Kethllen Stephanie Beranger, Edna Linhares Garcia

**Affiliations:** 1 Universidade de Santa Cruz do Sul, Programa de Pós-Graduação em Promoção da Saúde, Santa Cruz do Sul, RS, Brasil

**Keywords:** Acceso a los Servicios de Salud, Cirugía de Reasignación de Sexo, Itinerario Terapéutico, Sistema Único de Salud, Revisión de Alcance, Access to Health Services, Gender-Affirming Surgery, Therapeutic Itinerary, Brazilian National Health System, Scoping Review

## Abstract

**Objective:**

To analyze the representation of access pathways to the transsexualizing process in Brazil and the main barriers faced by transgender people.

**Method:**

A scoping review of the literature was conducted. Searches took place in January 2024, with articles and reviews that addressed access to the transsexualizing process being eligible, while books, chapters, conferences, editorial documents, and studies describing surgical procedures were excluded.

**Results:**

A total of 776 articles were found, of which 9 were included. 66% of the studies analyzed were field research involving interviews. The findings highlight the 5 stages of the transsexualizing process: care at primary healthcare centers, referral, multidisciplinary follow-up, surgical procedure, and local care for individuals.

**Conclusion:**

The studies focused on the barriers to accessing the transsexualizing process, rather than on the access pathways themselves, highlighting a gap in the understanding of the access itineraries to the process. rather than on the access pathways themselves, highlighting a gap in understanding the itineraries to the process.

## Study contributions

**Main results:**

Discrimination and prejudice permeate the pathways taken by trans people to access the transsexualizing process. Related studies are limited and primarily focus on the challenges rather than on the routes to access.

**Implications for services:**

Presenting the access pathways to the transsexualizing process in the Brazilian National Health System (SUS) could contribute to more accurate and appropriate referrals within healthcare services.

**Perspectives:**

Future research should explore the transsexualizing process in practice and support the development of public policies aimed at providing comprehensive and humane care for transgender people.

## RESUMEN

**Objetivo:**

Analizar la representación de los itinerarios de acceso al proceso de atención transexualizadora en Brasil y las principales barreras enfrentadas por las personas trans.

**Método:**

Se realizó una revisión de alcance de la literatura. Las búsquedas se llevaron a cabo en enero de 2024, siendo elegibles los artículos y revisiones que abordaban el acceso al proceso de atención transexualizadora, mientras que se excluyeron libros, capítulos, conferencias, documentos editoriales y estudios que describían procedimientos quirúrgicos.

**Resultados:**

Se localizaron 776 artículos, de los cuales se incluyeron 9. El 66% de los trabajos analizados fueron investigaciones de campo basadas en entrevistas. Los hallazgos evidencian las 5 etapas del proceso de atención transexualizadora: atención en unidades básicas de salud, derivación, seguimiento multidisciplinario, procedimiento quirúrgico y atención local a los individuos.

**Conclusión:**

Los estudios se centraron en las barreras de acceso al proceso de atención transexualizadora, y no en las formas de acceso, evidenciando una brecha en la comprensión de los itinerarios de acceso al proceso.

## INTRODUCTION

Historically, transsexuality has been considered as a deviation from the binary gender norm, resulting in stigmatization and denial of rights for transgender people (including transvestites, transsexuals, transgender individuals, transgender women and men).^1,2^ Since the beginning of the 21^st^ century, the pathological biomedical view has been contested, with the human rights paradigm bringing greater visibility and increasing research on the topic. The current focus is on defending the legal and social rights of these people, with emphasis on violations such as difficulties in accessing healthcare, employment, education, privacy and safety.^3^

In Brazil, data on the transgender population are limited. Transgender people represented 0.69% of the population and non-binary people, 1.19%, in a survey conducted in 2021, totaling nearly 3 million people.^4^ Two-thirds were not in a relationship, and 85% of transgender men and 50% of transgender women faced challenges with gender-related body characteristics.^4^ Nearly all had completed up to high school and 4% had higher education. Economically, 44.6% belonged to classes D and E, and 38.1% to class C, reflecting the employment challenges faced by this vulnerable population.^4^

Over the past seven years, the National Association of Transvestites and Transsexuals recorded 1,057 homicides of transgender people, transvestites and non-binary people in Brazil. In 2023, there were 145 homicides – an increase of 10.7% compared to 2022 (131 homicides).^5^ In 2023, murders of this population in Brazil increased by 150% compared to 2008. Seventy-nine percent of the victims were under 35 years old and many were engaged in sex work. The overall homicide rate decreased by 5.7%. Transvestites and transgender women are up to 32 times more likely to be murdered compared to transgender men and transmasculine individuals.^5^ In 2023, 94% of the murders of transgender people worldwide were of transgender women or transfeminine people.^6^

The high murder rate may reflect both an actual increase and an increase in records. Data are underreported, many records are inadequate, and there is a lack of training among the responsible agencies.^7^ In Europe, there were 12 homicides of transgender people in the past five years, and in Africa, 18 between 2008 and 2020. In countries such as Afghanistan and Uzbekistan, where being trans is a serious crime, there was only one homicide recorded in the same period.^5^

Progress in public policies has been slow. In 2008, the Ministry of Health issued Ordinance No. 457 authorizing the transsexualizing process within the Brazilian National Health System (*Sistema Único de Saúde* - SUS). In 2011, the National Policy for Comprehensive Health for Lesbians, Gays, Bisexuals, Transvestites and Transsexuals was established through Ordinance No. 2,836. In 2013, Ordinance No. 2,803 was created, revoking Ordinance No. 1,707/2008, in order to redefine and expand the transsexualizing process within the SUS. This ordinance aims to integrate care for transgender people, extending beyond just gender-affirming surgeries. It proposed interdisciplinary work, humanized and non-discriminatory care, and respect for human diversity.^8^

Recent legislation represents progress in ensuring the right to health for the transgender population. Monitoring for the transsexualizing process began in 2018, requiring psychiatric reports, psychological, gynecological, urological, endocrine and plastic surgery evaluations, in addition to regulating deadlines for hormonal or surgical interventions.^9^

Transgender people face various challenges in accessing health care. Examples include discrimination, the pathologization of transsexuality, inadequate care, the requirement of surgery for name and gender change, lack of professional qualifications, absence of primary care policies and scarcity of resources.^10^ Many healthcare professionals impose their personal beliefs and moral values over the rights of patients.^11^

Despite legislation, access to services and the elimination of discrimination have not been fully achieved, as this requires a social and cultural change that recognizes diversity beyond the binary gender norm. The lack of information about the transsexualizing process remains a significant challenge.^12^

Understanding the pathways transgender people take to access the transsexualizing process within the SUS is crucial, as it reveals their particularities and the diversity of care and social and cultural access.^13^ This study aimed to analyze these pathways and identify the main barriers they face.

## METHOD

A scoping literature review was conducted^14^ following seven steps: elaboration of the research problem and inclusion and exclusion criteria; database search; data extraction and organization in an Excel spreadsheet; reading of titles and abstracts; reading of selected articles and assessment of methodological quality; synthesis of results; assessment of evidence and its quality; and drafting of results and discussion.^15^

The population, intervention, comparison, outcome and context (PICOC) framework was used to define the research question: What are the pathways taken by transgender people to access the transsexualizing process within the SUS? The components of the research question are shown in Box 1.

**Box 1:** Components of the research question

Elements	Description
**Population**	Studies on *travestis*, transgender women, transgender men, transgender people, transsexuals, non-binary people, healthcare professionals, health managers.
**Intervention**	Health policies and programs aimed at the LGBTQIAPN+ population, organization of healthcare services.
**Comparison**	Not applicable.
**Results**	Different access routes to the transsexualizing process within the Brazilian National Health System (*Sistema Único de Saúde* - SUS).
**Context**	Specialized healthcare services for trans individuals in the SUS.

The search terms were defined based on the main and alternative terms of the Health Sciences Descriptors and the Medical Subject Headings (MeSH). Although “transsexualizing process” is not a descriptor in the health sciences, it was included in the search as it is the official nomenclature used in Brazilian public policy. Data exportation took place on January 1, 2024, from the PubMed, Scientific Electronic Library Online (SciELO), Scopus, and Web of Science Core Collection databases.

In the Scopus and Web of Science Core Collection databases, the following search terms were used: ((“sex reassignment surgery” OR “sex reassignment procedures” OR “sexual suitability” OR “sex appropriateness” OR “gender affirming” OR “gender confirmation” OR “genital reconstruction” OR “genital reassignment” OR “sexual reassignment” OR “gender reassignment” OR “sex reassignment” OR “revitalization” OR “sex change” OR “transsexualism process”) AND (“Brazil” OR “Unified Health System” OR “ SUS”) AND “access”). The search was refined by country (Brazil) and types of documents (articles and reviews).

In PubMed , the initial searches included hundreds of articles unrelated to the study topic. Health science descriptors were used to define the terms for the searches in Web of Science and Scopus. MeSH terms were used for the search in PubMed , with the latter using the following search terms: ((“transsexualizing process” OR “sex reassignment surgery” OR “sex reassignment procedures”) AND “Brazil”). No filters were used for categories, topics, research areas, languages and year of publication. The same search terms were used for SciELO. All results were imported into Microsoft Excel to remove duplicates.

Inclusion criteria were: discussing access to the transsexualizing process; referring to the trans, transsexual or transgender population in the Brazilian context; and being either an original article or a systematic review, published on any date and in any language. An article was included by searching the references of eligible studies. The exclusion criteria included chapters, conferences, books, letters to the editor, editorial documents and clinical articles describing surgical procedures that did not undergo ethical review or failed to present consistent methodological steps, such as inadequate data collection or omission of methodological procedures.

After defining the problem, objective and scope of the research, the PRISMA Extension for Scoping Reviews (PRISMA- ScR ) was used,^16^ and all steps were performed by two independent researchers and concomitantly, including the search process (MSB and IP), data collection (MSB and IP) and reading and classification of articles (MSB and IP). In cases where there were doubts or disagreements between the researchers, a third author was consulted to establish a consensus.

The data were summarized based on the results of the studies, divided into two categories: stages of the transsexualizing process and access barriers. No meta-analysis was performed due to the descriptive nature of the data. Drafting and discussion of the results were conducted by all authors (MSB, IP, KSB and ELG). This study was not evaluated by a research ethics committee, as it used only secondary data from published studies. As this was a scoping review, there was no bias analysis of the included studies.^17^ Confidence in qualitative results was not evaluated, as the focus was on the stages of the transsexualizing process.

From the included studies, a thematic network was generated, consisting of a keyword co-occurrence network. The network was generated using the VosViewer program,^18^ in which spheres represent the themes that stood out most in the field of study. The size of the sphere (theme) was proportional to the number of associated documents, and the lines represented the relationships between the themes. The more lines originating from a theme, the greater the centrality of that theme in the field of study.^18^ The themes were grouped by colors. with clusters representing the association and co-occurrence of themes over time.^18^

## RESULTS

**Figure 1:**
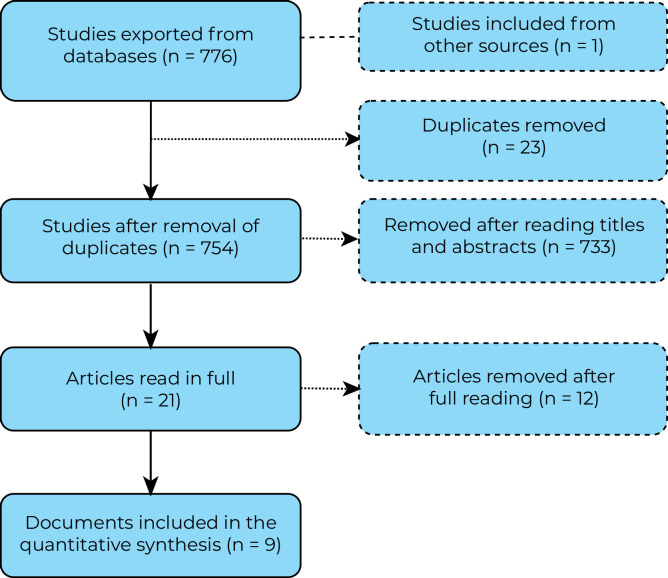
Process of including studies in the scoping review

A total of 776 publications were identified, including 84 in PubMed, 13 in Scopus and 679 in Web of Science (Figure 1). Nine studies were included in the descriptive synthesis (Table 1). Supplementary Table 1 provides additional data, such as the objective of the articles analyzed and the barriers and difficulties in accessing the transsexualizing process, limitations and recommendations.

**Table 1:** Main characteristics of the studies included in the review (n=9)

Study	Study design	Location	Participants (n)	Stages of the transsexualizing process
Costa AB, Rosa Filho HT, Pase PF, Fontanari AMV, Catelan RF, Mueller A, et al. 2018^21^	Cross-sectional hospital-based study.	Rio Grande do Sul and São Paulo	626	4
Hanauer OF D, Hemmi APA. 2019^27^	Qualitative, exploratory field research, interviews.	Minas Gerais	7	1
Lima RRT, Flor TBM, Noro LRA. 2023^20^	Systematic review.	N/A	N/A	1
Mattos MH, Zambenedetti G. 2021^23^	Qualitative research, analytical-institutional, interviews.	Northern region of Brazil	18	1,2,3,4
Monteiro S, Brigeiro M. 2019^22^	Qualitative field research, interviews.	Metropolitan Region of Rio de Janeiro	9	1,2,3,4,5
Popadiuk GS, Oliveira DC, Signorelli MC. 2017^19^	Quantitative-qualitative research, with document analysis.	Brazil	N/A	1,2,4
Rocon PC, Sodre F, Rodrigues A, Barros MEB de, Wandekoken KD. 2019^24^	Qualitative field research, interviews.	Southeast region of Brazil	9	4
Santos MOF, Olivar JMN. 2020^9^	Ethnographic field research, interviews.	São Paulo	2	1
Silva RCD, Silva ABB, Alves, FC, Ferreira KG, Nascimento LDV, Alves MF, et al. 2022^25^	Integrative review.	N/A	N/A	1,2,3

N/A – Not applicable as these are document analysis or review studies.

**Figure 2:**
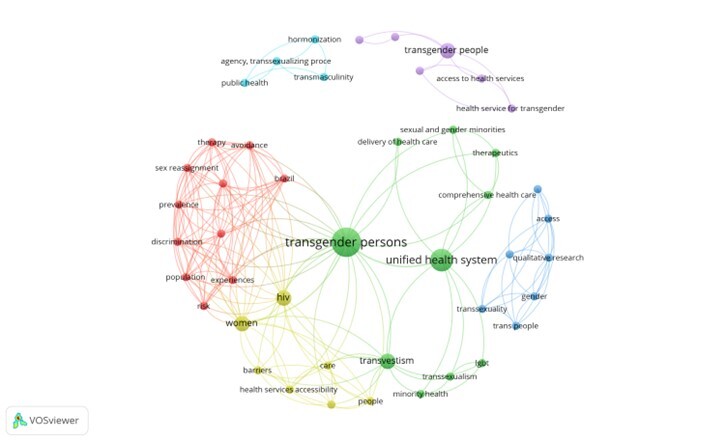
Network analysis of themes based on keyword co-occurrence

The most prominent themes were “transgender people” and “SUS”, representing the core focus of the research. The “transgender people” network was related to HIV, discrimination, sexual reassignment and therapy, among others, highlighting the most addressed topics in the studies (Figure 2). The relationship between transgender people and SUS revealed themes such as healthcare access, integrated care, care delivery, and minorities.

**Figure 3:**
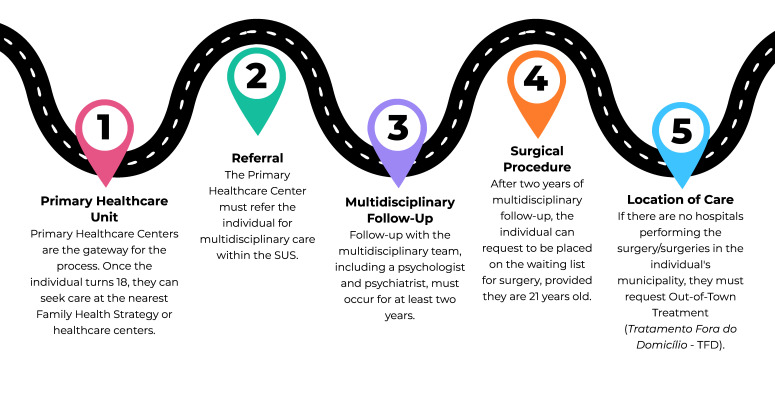
Access pathways to the transsexualization process within the Brazilian National Health System

The identification of the main themes (Figure 2) helped in understanding the development of the subject and to determine which topics are more advanced or require further research. The narrative synthesis showed the results of the thematic network and the interfaces between the themes. The barriers to access the transsexualizing process and the need for a multidisciplinary approach in gender identity studies were highlighted. Through this analysis, it was possible to identify the itinerary of the transsexualizing process. Figure 3 presents and describes the stages of this process. It begins with the primary healthcare centers (stage 1) which initiate the process and refer people (stage 2) for multidisciplinary follow-up within SUS (stage 3). After two years of follow-up, stage 4 can be initiated, which consists of the request for surgical procedures. Stage 5 refers to the place of care for individuals.

Studies report that, in order to implement the transsexualizing process within the SUS, it is not enough to establish guidelines for care, but rather to reclaim the principles of comprehensiveness and universality of the SUS. The goal is to have a set of actions that ensure the right to healthcare, especially in the context of specialized care.^19^ The implementation of the transsexualizing process within the SUS represents a significant advancement in promoting the health of transgender people.^20^

## DISCUSSION

The findings of this study, based on the literature analyzed, present the five stages of the SUS transsexualizing process: primary healthcare services, referral, multidisciplinary follow-up, surgery request, and local healthcare provision. Disrespect for the use of the social name, ridicule and discrimination faced by transgender people in health services were also observed in the studies,^21,25^ advocating the need for specific training for healthcare professionals, aiming to combat discrimination and improve care delivery.^19,21^

This study has limitations, such as the restricted databases and the small sample size, which prevent the generalization of the results. The observational nature does not allow for determining causality or performing statistical analyses, and the scoping review method offers a descriptive view without meta-analytic generalizations. Despite the independent analysis by two researchers, there is a risk of bias, especially in qualitative studies. In addition, it was identified that many of the studies analyzed do not offer practical solutions for transgender people, failing to fulfill the role of science in disseminating applicable and relevant knowledge for all.

Although the transsexualizing process has been in place for 10 years, information about the access pathways remains limited. In general, studies focus on the challenges, rather than access pathways.

The therapeutic itinerary for transgender people included two components: primary care and specialized care. Primary care serves as the gateway to the health system, responsible for providing care and referrals to specialists. Specialized care includes outpatient care (hormone therapy and psychotherapeutic follow-up) and hospital care (sexual reassignment surgeries and pre- and post-operative care).^28^

Outpatient care in Brazil is provided by six specialized outpatient clinics for the care of the transgender population within the SUS: Research and Support Center for Transvestites and Transsexuals of Curitiba, Instituto Estadual de Diabetes e Endocrinologia Luiz Capriglione do Rio de Janeiro, Hospital Universitário Professor Edgard Santos de Salvador, Centro de Referência e Treinamento DST/AIDS de São Paulo, ambulatório do Hospital das Clínicas de Uberlândia e Hospital Universitário Cassiano Antônio de Moraes de Vitória. There are also state outpatient clinics.^29^

In the fourth stage of the transsexualizing process, which involves surgical procedures, hospital care must be provided at one of the five hospitals accredited to perform the transsexualizing process, namely: Hospital das Clínicas da Universidade Federal de Goiás, Hospital de Clínicas de Porto Alegre, Hospital das Clínicas da Universidade Federal de Pernambuco, Hospital Universitário Pedro Ernesto do Rio de Janeiro e Hospital de Clínicas da Universidade de São Paulo.^28^

Breast implants and genital surgery are the most sought-after procedures by transgender women and transvestites. Transgender men most frequently seek mastectomy (breast removal for masculinization) and hysterectomy (removal of the uterus). Phalloplasty surgery is still performed experimentally in Brazil.^28^ Sexual reassignment surgery is positioned at the end of a long healthcare journey through public services.

The stages of the transsexualizing process within the SUS are permeated by ignorance, neglect, exclusion and prejudice.^21^ The main obstacles to accessing the transsexualizing process are bureaucracy, long waiting times,^22^ the centralization of specialized services in large urban centers, which makes the geographic issue a social marker of difference,^23,24^ the pathologization demonstrated by healthcare professionals and the requirement for a gender identity disorder diagnosis.^25^

However, access barriers often lead transgender individuals to develop self-care and self-medication strategies that can put them at risk.^21-23^ When seeking for hormone therapy, transgender people face resistance from some professionals and healthcare services. The reasons for this are the lack of specialization, a failure to understand different gender identities, and denial of the referred identity. These experiences keep transgender people away from institutionalized health services.^26^

YouTube and Facebook and WhatsApp groups have proven to be the main sources of recommendations on medications, procedures and services for transgender people, as well as for information about transgender identity and possible body changes. The search for information online is a common feature of the therapeutic pathway for the majority of this population.^27^ Regional discrepancies in access to SUS services are another significant issue in the transsexualizing process. Previous studies have heavily criticized the geographical concentration and limitation of healthcare services.^24,25^

The concentration of specialized services in a few metropolitan regions violates the principle of universality of the SUS and creates geographic inequalities.^25^ Transgender individuals living in peripheral or rural areas face barriers to access and lack the financial resources for transportation. This prevents equitable access to health care for the most disadvantaged.

The persistence of barriers to accessing the transsexualizing process is partly due to the lack of adequate training for healthcare professionals^22,26^ The lack of continuing education on gender diversity perpetuates these barriers. This undermines the application of the principles of comprehensiveness and universality in the SUS.

This reality drives transgender people away from services and contributes to additional barriers, such as the pathologization of transgender identities and the requirement for gender identity disorder diagnoses as part of the transsexualizing process.^10^ Despite the progress represented by the transsexualizing process,^28^ limitations such as the implementation of inclusive public policies and inadequate dissemination of access pathways hinder its reach and effectiveness. The lack of direct involvement of the transgender population in policy formulation may also contribute to the persistence of these barriers.

This study contributes to the understanding of the transsexualizing process and suggests updating legislation and dissemination strategies, addressing an important gap. This makes it easier for transgender people to access their rights and provides guidance to healthcare professionals. It is crucial to adopt approaches committed to human rights and equity in healthcare delivery. The collection of sociodemographic data should include transgender people to avoid their invisibility. In addition, it is essential that transgender people participate in the formulation and reformulation of policies related to them, as they are most familiar with their own needs and particularities.

Future research should consider other databases and larger samples in an attempt to produce generalizable results. A longitudinal analysis could be conducted to explore the relationship between specific interventions and health outcomes for the transgender population. Empirical studies, such as case studies and observational studies, are suggested to understand the transsexualizing process in practice. This could involve analyses of SUS facilities, such as primary healthcare centers, as well as issues related to individuals starting the transsexualizing process. Other studies could delve deeper into the formulation of public policies aimed at reducing the challenges and difficulties associated with the process.

The stages of the transsexualizing process within the SUS lack transparency to provide humane care to individuals. Although the transsexualizing process consists of five defined stages, both healthcare professionals and patients have doubts about the process, which reinforces the importance of adequate professional training and the dissemination of information about the available services.

## Supplementary Table 1

**Table:** Summary of articles (n=9) included in the review

No.	Objective of the study	Barriers and challenges identified	Study limitations	Study recommendations
1	To analyze how the Ministry of Health has been implementing the Transsexualizing Process in the SUS.	Desrrespect for the social name and its legitimacy, division by sex and procedures considered feminine or masculine.	The study does not analyze individual data, focusing only on Ministry of Health documents.	Creation of training actions to combat discrimination based on gender, sexual orientation, race, color, ethnicity and territory for healthcare professionals, tools for evaluating and monitoring the transsexualizing process in all its stages, seeking to reduce prejudice.
2	To understand the specific healthcare needs of the transgender population and the access barriers they face.	Inadequate use of social name and pronoun. Lack of knowledge about rights and procedures available through the SUS.	Focused on individuals from only two states in the country whose educational level was above the Brazilian average, however, individuals with lower levels of education may face greater challenges in accessing health services.	Disseminate information to trans individuals about their rights through the SUS; create training courses for health professionals and distribute legal and administrative mechanisms to combat and prevent discrimination in the healthcare context.
3	To describe the pathways taken by transsexuals, aiming to understand their itineraries in the search for care for their health demands.	Lack of family and social support to initiate the transsexualizing process and long waiting time. Limited information about procedures and access to public services.	Low number of interviews conducted in only one state . No analysis categories were presented for the results. It is impossible to generalize the results.	Need for political action against exclusion and discrimination. Development of social, political and health strategies.
4	To analyze the experiences of trans/transvestite women in accessing health services and discuss sexual/gender discrimination in gender transition and AIDS prevention services.	Failure to respect the use of social names and pronouns . Services that do not fully meet the needs of individuals.	Low number of interviews. Impossible to generalize the results. No analysis categories were developed.	Training for health professionals. Improvements in the SUS operations and recognition of the specificities of each gender technology and how they interact, without assigning a different moral value to those managed by trans people themselves.
5	To analyze the challenges to universalizing access to the SUS transsexualizing process.	Discrimination and humiliation by health professionals. Disrespect for social name and pronouns .	Small number of individuals interviewed in a specific region with more advanced healthcare services. Impossibility of generalizing the results.	Rethink access to the transsexualizing process in the SUS and its requirements; geographically expand the transsexualization process within the SUS.
6	To analyze the pathway that trans men take to access the transsexualizing process in a public care center.	Long waiting times pelos for procedures. Communication problems between the different levels of care in the transsexualizing process. Lack of professional training absence of public policies that support rights of trans people.	Low number of interviews. Impossibility of generalizing the results. Absence of data triangulation. Analysis conducted with individuals from only one region of the state. The study is also limited to constructing the pathway based only on the participants’ narratives without additional validation.	Reduction in waiting times for procedures carried out by the SUS is necessary to provide quality of life for individuals and reduce the number of people seeking clandestine services. Training for health professionals.
7	To identify the difficulties that the trans population faces in accessing the SUS.	Geographic concentration of services; Prejudice and lack of knowledge among health professionals . Persistence of the pathologization of transsexuality.	Low number of studies on the trans population, mainly due to the lack of data and the limited research on the subject in Brazil.	Training healthcare professionals and create magazines, pamphlets, and materials that include the trans population, available in waiting rooms for information and inclusion. Allow trans individuals to choose which bathroom they wish to use.
8	To synthesize evidence on health care for transvestites and transsexuals in Brazil.	Lack of knowledge and prejudiced attitudes among healthcare professionals. . Disrespect for the use of the social name.	Limited number of studies included . Studies conducted in the northern region of the country were not included. Articles that analyzed access to healthcare for the trans population together with other LGBTQIA+ groups were excluded.	Understand current public policies and create new ones that protect the rights of trans people. Develop a health care model that integrates different aspects of care and mitigates prejudice in health serviices. Create ways to reduce the existing stigma about trans people and psychological problems.
9	To identify factors involved in hormone therapy for transmasculine individuals, from a perspective of agency and care.	Disrespect and lack of knowledge among health professionals. Limited number of specialized services. Pathologization of transsexuality.	The ethnographic analysis is based only on the observation of two transmasculine people . It is impossible to generalize the results of the study.	Inform about the multiple ways of being trans and transmasculine. Reevaluate self-medication from a perspective of agency and care. Rethink medical training to better prepare professionals to participate in the transsexualizing process.

N/A – Not applicable or not reported in the study.
