# Supplementary material for: Access pathways to the transsexualizing process in Brazil: a scoping review
Source: Epidemiol Serv Saude. 2025 Jan 10;33(spe1):e2024311. doi: 10.1590/S2237-96222024v33e2024311.especial.en (PMC11734662; doi:10.1590/S2237-96222024v33e2024311.especial.en)
Supplement: Translation

# Translation: Itinerários de acesso ao processo transexualizador no Brasil: uma revisão de escopo

**DOI:** 10.1590/S2237-96222024v33e2024311.especial.pt

**Authors:** Mariluza Sott Bender, Ingre Paz, Kethllen Stephanie Beranger, Edna Linhares Garcia

**Affiliations:** 1 Universidade de Santa Cruz do Sul, Programa de Pós-Graduação em Promoção da Saúde, Santa Cruz do Sul, RS, Brasil

**Keywords:** Acesso aos Serviços de Saúde, Cirurgia de Readequação Sexual, Itinerário Terapêutico, Sistema Único de Saúde, Revisão de Escopo

## Abstract

**Objetivo:**

Analisar a representação dos itinerários de acesso ao processo transexualizador no Brasil e as principais barreiras enfrentadas pelas pessoas trans.

**Método:**

Realizou-se revisão de escopo da literatura. As buscas ocorreram em janeiro de 2024, sendo elegíveis artigos e revisões que abordam o acesso ao processo transexualizador e excluídos livros, capítulos, conferências, documentos editoriais e estudos que descreviam procedimentos cirúrgicos.

**Resultados:**

Foram localizados 776 artigos, dos quais 9 foram incluídos. 66% dos trabalhos analisados foram pesquisas de campo com entrevistas. Os achados evidenciam as 5 etapas do processo transexualizador: atendimento nas unidades básicas de saúde, encaminhamento, acompanhamento multidisciplinar, procedimento cirúrgico e atendimento local aos indivíduos.

**Conclusão:**

Os estudos centravam-se nas barreiras de acesso ao processo transexualizador, e não nas formas de acesso, evidenciando lacuna sobre os itinerários de acesso ao processo.

## Contribuições do estudo

**Principais resultados:**

Discriminação e preconceito atravessam os itinerários das pessoas trans para acesso ao processo transexualizador. Os estudos relacionados são incipientes e centram-se principalmente nas dificuldades, e não nas formas de acesso.

**Implicações para os serviços:**

A apresentação dos itinerários de acesso ao processo transexualizador do Sistema Único de Saúde poderá contribuir com os serviços de saúde para encaminhamentos mais assertivos e adequados.

**Perspectivas:**

Pesquisas futuras devem investigar o processo de transexualização na prática e auxiliar na formulação de políticas públicas para o atendimento integral e humanizado das pessoas trans.

## INTRODUÇÃO

Historicamente, a transexualidade foi vista como um desvio da norma binária de gênero, resultando em estigmatização e negação de direitos das pessoas trans (incluindo travestis, transexuais, transgêneros, mulheres e homens trans).^1,2^ Desde o início do século XXI, a visão biomédica patológica tem sido contestada, com o paradigma dos direitos humanos trazendo mais visibilidade e aumentando a pesquisa sobre o tema. O foco atual é a defesa dos direitos jurídicos e sociais dessas pessoas, com destaque para violações como dificuldades no acesso a saúde, emprego, educação, privacidade e segurança.^3^

No Brasil, os dados sobre a população trans são limitados. Indivíduos trans representavam 0,69% da população e não binários, 1,19%, em levantamento realizado em 2021, totalizando quase 3 milhões de pessoas.^4^ Dois terços não estavam em relacionamento, e 85% dos homens e 50% das mulheres trans enfrentavam dificuldades com características corporais relacionadas ao gênero.^4^ A quase totalidade tinha até o ensino médio completo e 4%, ensino superior. Economicamente, 44,6% pertenciam às classes D e E, e 38,1%, na classe C, refletindo as dificuldades de inserção no mercado de trabalho nesta população vulnerabilizada.^4^

Nos últimos sete anos, a Associação Nacional de Travestis e Transexuais registrou 1.057 assassinatos de pessoas trans, travestis e não binárias no Brasil. Em 2023 foram 145 assassinatos – aumento de 10,7% em relação a 2022 (131 homicídios).^5^ Em 2023, os assassinatos desse grupo populacional no Brasil aumentaram 150% em relação a 2008. Setenta e nove por cento das vítimas tinham menos de 35 anos e muitas trabalhavam com prostituição. A taxa geral de homicídios caiu 5,7%. Travestis e mulheres trans têm até 32 vezes mais chances de serem assassinadas se comparadas a homens trans e pessoas transmasculinas.^5^ Em 2023, 94% dos assassinatos de pessoas trans no mundo foram de mulheres trans ou pessoas transfemininas.^6^

O alto índice de assassinatos pode refletir o aumento real e o aumento nos registros. Os dados estão subnotificados, muitos registros são inadequados e falta capacitação dos órgãos responsáveis.^7^ Na Europa, houve 12 homicídios de pessoas trans nos últimos cinco anos, e na África, 18 entre 2008 e 2020. Em países como Afeganistão e Uzbequistão, onde ser trans é um crime grave, houve apenas um assassinato registrado no mesmo período.^5^

Os avanços em políticas públicas são lentos. Em 2008, o Ministério da Saúde publicou a Portaria nº 457 autorizando o processo transexualizador no Sistema Único de Saúde (SUS). Em 2011, foi criada a Política Nacional de Saúde Integral de Lésbicas, *Gays*, Bissexuais, Travestis e Transexuais, com a Portaria nº 2.836. Em 2013, foi criada a Portaria nº 2.803 que revogou a Portaria nº 1.707/2008, a fim de redefinir e ampliar o processo transexualizador no SUS. Essa portaria objetiva a integração dos atendimentos às pessoas trans, sem restringi-los às cirurgias de redesignação sexual. Propõe o trabalho interdisciplinar, o acolhimento humanizado e sem discriminação e o respeito pelas diferenças humanas.^8^

As legislações recentes representam um avanço no direito à saúde da população trans. O acompanhamento para o processo transexualizador começou em 2018, exigindo laudos psiquiátricos, avaliações psicológicas, ginecológicas, urológicas, endocrinológicas e plásticas, além de regulamentar prazos para intervenções hormonais ou cirúrgicas.^9^

As pessoas trans enfrentam várias dificuldades no atendimento à saúde. São exemplos de dificuldades: discriminação, patologização da transexualidade, acolhimento inadequado, exigência de cirurgia para troca de nome e sexo, falta de qualificação dos profissionais, ausência de políticas de atenção básica e escassez de recursos.^10^ Muitos profissionais de saúde sobrepõem suas crenças e valores morais aos direitos dos pacientes.^11^

Apesar das legislações, o acesso aos serviços e a eliminação da discriminação não foram garantidos, pois isso requer uma mudança social e cultural que reconheça a diversidade além da norma binária. A falta de informação sobre o processo transexualizador é uma dificuldade significativa.^12^

Conhecer os itinerários das pessoas trans para acessar o processo transexualizador no SUS é crucial, visto que revela suas particularidades e as diversidades de cuidados e acessos sociais e culturais.^13^ Este estudo objetivou analisar esses itinerários e identificar as principais barreiras enfrentadas.

## MÉTODO

Realizou-se uma revisão de escopo da literatura^14^ que seguiu sete etapas: elaboração do problema de pesquisa e dos critérios de inclusão e exclusão; pesquisa nas bases de dados; extração dos dados e organização em planilha de Excel; leitura dos títulos e resumos; leitura dos artigos selecionados e avaliação da qualidade metodológica; realização da síntese dos resultados; avaliação das evidências e sua qualidade; e redação dos resultados e discussão.^15^

A estrutura população, intervenção, comparação, resultado e contexto (PICOC) foi utilizada para delimitação da pergunta de pesquisa: Quais os itinerários percorridos pelas pessoas trans para acessar o processo transexualizador do SUS? Os componentes da pergunta de pesquisa são apresentados no Quadro 1.

**Quadro 1:** Componentes da pergunta de pesquisa

Elementos	Descrição
**População**	Estudos sobre travestis, mulheres trans, homens trans, transgêneros, transexuais, não binários, profissionais da saúde, gestores da saúde.
**Intervenção**	Políticas e programas de saúde voltados à população LGBTQIAPN+, organização dos serviços de saúde.
**Comparação**	Não se aplica.
**Resultados**	Diferentes itinerários de acesso ao processo transexualizador no Sistema Único de Saúde (SUS).
**Contexto**	Serviços de saúde especializados em atendimentos a pessoas trans do SUS.

Os termos de busca foram definidos a partir dos termos principais e alternativos dos Descritores em Ciências da Saúde e do Medical Subject Headings (MeSH). Apesar de os descritores em ciências da saúde não apresentarem o termo transsexualizing process como descritor, esse foi incluído nas buscas por ser a nomenclatura oficial utilizada na política pública brasileira. A exportação dos dados ocorreu em 1º de janeiro de 2024 nas bases de dados PubMed, Scientific Electronic Library Online, Scopus e Web of Science Core Collection. 

Nas bases Scopus e Web of Science Core Collection, utilizou-se os seguintes termos de busca: ((“sex reassignment surgery” OR “sex reassignment procedures” OR “sexual suitability” OR “sex appropriateness” OR “gender affirming” OR “gender confirmation” OR “genital reconstruction” OR “genital reassignment” OR “sexual reassignment” OR “gender reassignment” OR “sex reassignment” OR “revitalization” OR “sex change” OR “transsexualism process”) AND (“Brazil” OR “Unified Health System” OR “SUS”) AND “access”). Refinou-se a busca por país (Brasil) e tipos de documentos (artigos e revisões). 

Na PubMed, as buscas iniciais incluíam centenas de artigos não relacionados com a temática de estudo. Descritores em ciências da saúde foram utilizados para a definição dos termos para as buscas na Web of Science e na Scopus. Termos MeSH foram utilizados para a busca na PubMed, tendo nesta última utilizado os seguintes termos de busca: ((“transsexualizing process” OR “sex reassignment surgery” OR “sex reassignment procedures”) AND “Brazil”). Não foram utilizados filtros de categorias, tópicos, áreas de pesquisa, idiomas e ano de publicação. Na SciELO foram utilizados os mesmos termos de busca. Todos os resultados foram importados para Microsoft Excel para remoção de duplicações.

Os critérios de inclusão foram: discutir o acesso ao processo transexualizador; referir-se à população trans, transexual ou transgênero no contexto brasileiro; e ser artigo original ou de revisão sistemática da literatura, publicado em qualquer data e idioma. Um artigo foi incluído por meio de busca nas referências dos estudos elegíveis. Foram critérios de exclusão capítulos, conferências, livros, cartas ao editor, documentos editoriais e artigos clínicos que descreviam procedimentos cirúrgicos, não passaram por comitê de ética em pesquisa e não evidenciavam etapas metodológicas consistentes, com coleta de dados inadequada ou omissão de procedimentos metodológicos.

Após a definição do problema, objetivo e escopo da pesquisa, o PRISMA Extension for Scoping Reviews (PRISMA-ScR) foi utilizado,^16^ sendo que todos os passos foram realizados por dois pesquisadores independentes e de forma concomitante, incluindo o processo de busca (MSB e IP), a coleta de dados (MSB e IP) e a leitura e classificação dos artigos (MSB e IP). Nos casos em que ocorreram dúvidas ou discordância entre os pesquisadores, um terceiro autor foi acionado para estabelecer um consenso. 

Os dados foram sumarizados com base nos resultados dos estudos, divididos em duas categorias: etapas do processo transexualizador e dificuldades de acesso. Não foi realizada meta-análise devido à natureza descritiva dos dados. A escrita e discussão dos resultados foram conduzidas por todos os autores (MSB, IP, KSB e ELG). Este estudo não foi avaliado por comitê de ética em pesquisa, pois utilizou apenas dados secundários de estudos publicados. Por ser revisão de escopo, não houve análise de viés dos estudos incluídos.^17^ A confiança dos resultados qualitativos não foi avaliada, visto que se focou as etapas do processo transexualizador.

A partir dos estudos incluídos, foi gerada uma rede temática que consiste em uma rede de coocorrência de palavras-chave. A rede foi gerada com apoio do programa VosViewer,^18^ cujas esferas são formadas pelos temas que mais se destacaram no campo de estudo. O tamanho da esfera (tema) foi apresentado de forma proporcional ao número de documentos associados, e as linhas representam as relações entre os temas. Quanto maior o número de linhas que partem de um tema para outros, maior a centralidade desse tema no campo de estudo.^18^ Os temas foram agrupados por cores. Os agrupamentos representam a associação e a coocorrência entre os temas ao longo do tempo.^18^

## RESULTADOS

Foram identificadas 776 publicações, sendo 84 na PubMed, 13 na Scopus e 679 na Web of Science (Figura 1). Foram incluídos nove estudos na síntese descritiva (Tabela 1). A Tabela Suplementar 1 apresenta dados complementares, como o objetivo dos artigos analisados e as barreiras e dificuldades de acesso ao processo transexualizador, limitações e recomendações.

**Figura 1:** Processo de inclusão dos estudos na revisão de escopo

**Tabela 1:** Principaiscaracterísticas dos estudos incluídos na revisão (n=9)

Estudo	Delineamento	Local	Participantes (n)	Etapas do processo transexualizador
Costa AB, Rosa Filho HT, Pase PF, Fontanari AMV, Catelan RF, Mueller A, et al. 2018^21^,	Pesquisa transversal hospitalar.	Rio Grande do Sul e São Paulo	626	4
Hanauer OF D, Hemmi APA. 2019^27^,	Pesquisa de campo qualitativa, exploratória, entrevistas.	Minas Gerais	7	1
Lima RRT, Flor TBM, Noro LRA. 2023^20^,	Revisão Sistemática.	N/A	N/A	1
Mattos MH, Zambenedetti G. 2021^23^,	Pesquisa qualitativa, analítico-institucional, entrevistas.	Região Norte do Brasil	18	1,2,3,4
Monteiro S, Brigeiro M. 2019^22^,	Pesquisa de campo qualitativa, entrevistas.	Região Metropolitana do Rio de Janeiro	9	1,2,3,4,5
Popadiuk GS, Oliveira DC, Signorelli MC. 2017^19^,	Pesquisa quanti-qualitativa, com análise documental.	Brasil	N/A	1,2,4
Rocon PC, Sodré F, Rodrigues A, Barros MEB de, Wandekoken KD. 2019^24^,	Pesquisa de campo qualitativa, entrevistas.	Região Sudeste do Brasil	9	4
Santos MOF, Olivar JMN. 2020^9^,	Pesquisa de campo etnográfica, entrevistas.	São Paulo	2	1
Silva RCD, Silva ABB, Alves, FC, Ferreira KG, Nascimento LDV, Alves MF, et al. 2022^25^,	Revisão Integrativa.	N/A	N/A	1,2,3

Os temas mais destacados foram “pessoas transgêneros” e “SUS”, representando a abordagem central da pesquisa. A rede “pessoas transgêneros” relacionou-se a HIV, discriminação, redesignação sexual e terapia, entre outros, evidenciando os assuntos mais abordados nos estudos (Figura 2). A relação entre pessoas trans e o SUS revelou temas como acesso à saúde, cuidados integrados, prestação de cuidados e minorias.

**Figura 2:** Análise de redes dos temas com base na coocorrência de palavras-chave

A identificação dos temas principais (Figura 2) ajudou a entender o desenvolvimento do assunto e a determinar quais tópicos estão mais avançados ou necessitam de mais pesquisa. A síntese narrativa mostrou os resultados da rede temática e as interfaces entre os temas. Destacaram-se as barreiras de acesso ao processo transexualizador e a necessidade de uma abordagem multidisciplinar nos estudos de identidade de gênero. Por meio da análise, foi possível identificar o itinerário do processo transexualizador. A Figura 3 apresenta e descreve as etapas do processo. Este se inicia pelas unidades básicas de saúde (etapa 1) que dão entrada ao processo e realizam o encaminhamento (etapa 2) para o acompanhamento multidisciplinar do SUS (etapa 3). Após dois anos de acompanhamento, pode ser iniciada a etapa 4, que consiste na solicitação para procedimento cirúrgico. A etapa 5 refere-se ao local de atendimento dos indivíduos. 

**Figura 3:** Itinerários de acesso ao processo transexualizador do Sistema Único de Saúde

Os estudos relatam que, para a implementação do processo transexualizador no SUS, não basta estabelecer diretrizes que norteiam o atendimento, mas sim resgatar os princípios de integralidade e universalidade do SUS. Tem-se como propósito a existência de conjunto de ações que garantam o direito à saúde, principalmente no contexto da atenção especializada.^19^ A implementação do processo transexualizador no SUS representa avanço significativo para a promoção da saúde das pessoas trans.^20^

## DISCUSSÃO

Os achados deste estudo, a partir da literatura analisada, apresentam as cinco etapas do processo transexualizador do SUS: o atendimento pelas unidades básicas de saúde, o encaminhamento, o acompanhamento multidisciplinar, a solicitação de cirurgia e o atendimento local dos indivíduos. O desrespeito ao uso do nome social, ridicularização e discriminação enfrentada pelas pessoas trans nos serviços de saúde também foi observadas nos estudos,^21,25^ defendendo a necessidade de formação específica para os profissionais de saúde, visando combater a discriminação e melhorar o atendimento.^19,21^

Este estudo tem limitações, como a restrição de bases de dados e a amostra pequena, que impedem a generalização dos resultados. A natureza observacional não permite determinar causalidade ou realizar análises estatísticas, e o método de revisão de escopo oferece uma visão descritiva sem generalizações meta-analíticas. Apesar da análise independente de dois pesquisadores, há risco de viés, especialmente em estudos qualitativos. Também foi identificado que muitas pesquisas analisadas não oferecem soluções práticas para as pessoas trans, não cumprindo o papel da ciência de disseminar conhecimento aplicável e relevante para todos.

Apesar de o processo transexualizador ter completado 10 anos, a divulgação sobre as formas de acesso é limitada. De forma geral, os estudos focam as dificuldades, e não as formas de acesso. 

O itinerário terapêutico das pessoas trans incluiu dois componentes: a atenção básica e a atenção especializada. A atenção básica é a porta de entrada para o sistema de saúde, responsável por atendimentos e encaminhamentos para especialidades. A atenção especializada abrange atendimento ambulatorial (hormonização e acompanhamento psicoterapêutico) e hospitalar (procedimentos cirúrgicos de redesignação sexual e acompanhamento pré e pós-operatório).^28^

O atendimento ambulatorial no Brasil é realizado por seis ambulatórios especializados para o atendimento da população trans do SUS: Centro de Pesquisa e Apoio a Travestis e Transexuais de Curitiba, Instituto Estadual de Diabetes e Endocrinologia Luiz Capriglione do Rio de Janeiro, Hospital Universitário Professor Edgard Santos de Salvador, Centro de Referência e Treinamento DST/AIDS de São Paulo, ambulatório do Hospital das Clínicas de Uberlândia e Hospital Universitário Cassiano Antônio de Moraes de Vitória. Também existem ambulatórios estaduais.^29^

Na quarta etapa do processo transexualiador, que refere aos procedimentos cirúrgicos, o atendimento hospitalar deve ser realizado em um dos cinco hospitais habilitados para realizar o processo transexualizador, a constar: Hospital das Clínicas da Universidade Federal de Goiás, Hospital de Clínicas de Porto Alegre, Hospital das Clínicas da Universidade Federal de Pernambuco, Hospital Universitário Pedro Ernesto do Rio de Janeiro e Hospital de Clínicas da Universidade de São Paulo.^28^

O implante de silicone nas mamas e a cirurgia genital são os procedimentos mais procurados pelas mulheres trans e travestis. Os homens trans procuram com maior frequência a mastectomia (retirada dos seios para masculinização) e a histerectomia (retirada do útero). A cirurgia de faloplastia ainda é realizada de forma experimental no Brasil.^28^ Identifica-se que o procedimento cirúrgico de redesignação sexual está no final de um longo itinerário pelos serviços de saúde.

As etapas do processo transexualizador pelo SUS são permeadas pelo desconhecimento, descaso, exclusão e preconceito.^21^ Os principais dificultadores do acesso ao processo transexualizador são a burocracia, o longo tempo de espera,^22^ a centralização dos serviços especializados nos grandes centros urbanos, o que torna a questão geográfica um marcador social da diferença,^23,24^ a patologização demonstrada pelos profissionais de saúde e a necessidade de um diagnóstico de transtorno de identidade de gênero.^25^

No entanto, as dificuldades de acesso fazem com que as pessoas trans construam trajetórias de autocuidado e automedicação que podem colocá-los em risco.^21-23^ Ao buscar atendimento para hormonização, as pessoas trans se deparam com a resistência de alguns profissionais e serviços de saúde. As justificativas para isso são a carência de especialização, a falta de compreensão das diferentes identidades de gênero existentes e a negação da identidade referida. Essas vivências afastam as pessoas trans dos serviços institucionalizados de saúde.^26^

O Youtube e grupos do Facebook e do WhatsApp revelaram-se como principal responsável pela indicação de medicamentos, procedimentos e serviços para as pessoas trans e pela compreensão sobre a transexualidade e as mudanças corporais possíveis. A busca de informações na internet compõe o itinerário da grande maioria dessa população.^27^ A discrepância regional no acesso aos serviços do SUS é outro ponto relevante no processo transexualizador. A concentração geográfica e a limitação dos serviços de saúde são fortemente criticadas em estudos prévios.^24,25^

Reitera-se que a concentração dos serviços especializados em poucas regiões metropolitanas é uma violação do princípio de universalidade do SUS e cria desigualdades geográficas.^25^ Pessoas trans residentes em áreas periféricas ou rurais enfrentam dificuldades de acesso e falta de recursos financeiros para deslocamento. Isso impede o acesso equitativo à saúde para os mais desfavorecidos. 

A persistência de barreiras no acesso ao processo transexualizador deve-se, em parte, à falta de treinamento adequado dos profissionais de saúde ^22,26^ A ausência de educação continuada sobre diversidade de gênero perpetua essas barreiras. Isso prejudica a aplicação dos princípios de integralidade e universalidade do SUS.

Essa realidade afasta as pessoas trans dos serviços e contribui para barreiras adicionais, como a patologização das identidades trans e a exigência de diagnósticos de transtorno de identidade de gênero para o processo transexualizador.^10^ Apesar do avanço representado pelo processo transexualizador,^28^ limitações como a efetivação de políticas públicas inclusivas e a divulgação inadequada das formas de acesso dificultam seu alcance e sua eficácia. A falta de envolvimento direto da população trans na formulação de políticas também pode contribuir para a persistência dessas barreiras.

Este estudo contribui para o entendimento do processo transexualizador e sugere a atualização de legislações e estratégias de divulgação, preenchendo uma lacuna importante. Isso facilita o acesso das pessoas trans a seus direitos e orienta os profissionais de saúde. É crucial adotar posturas comprometidas com os direitos humanos e a equidade nos atendimentos de saúde. A coleta de dados sociodemográficos deve incluir pessoas trans para evitar sua invisibilização. Também é essencial que as pessoas trans participem da formulação e reformulação das políticas relacionadas a elas, pois estas conhecem melhor suas necessidades e particularidades.

Pesquisas futuras devem considerar outras bases de dados e amostras maiores em tentativa de produzir generalizações dos resultados. Uma análise longitudinal pode ser realizada para explorar a relação entre intervenções específicas e resultados de saúde para a população trans. Sugere-se a realização de pesquisas empíricas, como estudos de caso e observacionais, para compreender o processo de transexualização na prática. Isso pode ser tanto por meio de análise dos estabelecimentos do SUS, como as unidades básicas de saúde, quanto por meio de questões relacionadas aos indivíduos que iniciam o processo de transexualização. Outros estudos podem abordar em profundidade a formulação de políticas públicas que auxiliem a reduzir os desafios e as dificuldades associadas ao processo.

As etapas do processo transexualizador pelo SUS carecem de transparência para proporcionar atendimento humano aos indivíduos. Embora o processo transexualizador possua cinco etapas definidas, tanto os profissionais de saúde quanto os pacientes possuem dúvidas sobre o processo, o que reforça a importância de capacitação adequada aos profissionais e divulgação das formas de atendimento ao público. 

## Tabela suplementar 1

**Table:** Síntese dos artigos (n=9) incluídos na revisão

Nº	Objetivo do estudo	Barreiras e dificuldades identificadas	Limitações do estudo	Recomendações do estudo
1	Analisar como o Ministério da Saúde vem implementando o Processo Transexualizador no SUS.	Desrespeito ao nome social e sua legitimidade, divisão por sexo e procedimentos considerados femininos ou masculinos.	O estudo não analisa dados de indivíduos, se debruçando apenas a documentos do Ministério da Saúde.	Criação de ações formativas de enfrentamento às discriminações de gênero, orientação sexual, raça, cor, etnia e território para profissionais da saúde e de ferramentas de avaliação e monitoramento do processo transexualizador em todas as suas etapas buscando diminuir o preconceito.
2	Compreender as necessidades específicas de cuidados de saúde da população trans e as barreiras de acesso enfrentadas.	Uso inadequado do nome social e do pronome. Desconhecimento sobre os direitos e procedimentos realizados pelo SUS.	Abordou indivíduos de apenas dois estados do país, cujo nível educacional era superior à média brasileira, no entanto, indivíduos com níveis de educação inferiores podem enfrentar desafios superiores no acesso ao serviço de saúde.	Difundir informação aos indivíduos trans sobre seus direitos por meio do SUS; criar cursos de formação aos profissionais da saúde e disseminar mecanismos legais e administrativos para coibir e prevenir a discriminação no contexto da saúde.
3	Descrever os caminhos percorridos por transexuais, visando conhecer seus itinerários na busca por atendimento às suas demandas em saúde.	Falta de apoio familiar e social para iniciar o processo transexualizador e longo tempo de espera. Limitadas informações sobre os procedimentos e acesso ao serviço público.	Baixo número de entrevistas realizadas em apenas um estado. Não foram apresentadas as categorias de análise dos resultados. Impossível a generalização dos resultados.	Necessidade de posicionamento político contra a exclusão e a discriminação. Desenvolvimento de estratégias sociais, políticas e de saúde.
4	Analisar as experiências de acesso de mulheres trans/travestis aos serviços de saúde e discutir a discriminação sexual/de gênero nos serviços de transição de gênero e prevenção da aids.	Não respeito ao uso do nome social e pronomes. Serviços que não atendem integralmente as necessidades dos indivíduos.	Baixo número de entrevistas. Impossível a generalização dos resultados. Não foram criadas categorias de análise.	Capacitação aos profissionais de saúde. Melhorias no funcionamento do SUS e reconhecimento das especificidades de cada tecnologia de gênero e como concorrem entre si, sem atribuir uma valoração moral diferenciada àquelas manejadas pelas próprias pessoas trans.
5	Analisar os desafios à universalização do acesso ao processo transexualizador do SUS.	Discriminação e humilhação por parte dos profissionais da saúde. Não respeito ao nome social e pronomes.	Pequeno número de indivíduos entrevistados em uma região específica, com maior avanço nos serviços de saúde. Impossibilidade de generalização dos resultados.	Repensar o acesso ao processo transexualizador do SUS e suas exigências; e expandir geograficamanete o processo transexualizador pelo SUS.
6	Analisar o percurso que homens trans percorrem para ter acesso ao processo transexualizador em um centro público de atenção e atendimento.	Longo tempo de espera pelos procedimentos. Problemas de comunicação entre os diferentes níveis de atendimento do processo transexualizador. Despreparo profissional e ausência de políticas públicas que amparem os direitos das pessoas trans.	Baixo número de entrevistas. Impossibilidade de generalização dos resultados. Ausência de triangulação dos dados. Análise com indivíduos de apenas uma região do estado. O estudo também se limita a construir o itinerário com base apenas nas narrativas dos participantes sem validação adicional.	Necessária redução no tempo de espera para realização de procedimentos pelo SUS para possibilitar qualidade de vida aos indivíduos e reduzir o número de buscas por serviços clandestinos. Formação para os profissionais de saúde.
7	Identificar as dificuldades que a população trans enfrenta para acessar o SUS.	Concentração geográfica dos serviços; Preconceito e desconhecimento dos profissionais de saúde. Persistência da patologização da transexualidade.	Pequeno número de estudos sobre a população trans, o que ocorreu principalmente devido à carência de dados e ao limitado número de pesquisas que abordam o assunto em território brasileiro.	Treinamento de profissionais da saúde e criação de revistas, panfletos e materiais que incluam a população trans e que sejam disponibilizados em salas de espera para informação e inclusão. Permitir que indivíduos trans escolham qual banheiro desejam utilizar.
8	Sintetizar evidências para caracterizar a atenção à saúde para travestis e transexuais no Brasil.	Desconhecimento e atitudes preceituosas e dos profissionais da saúde. Desrespeito ao uso do nome social.	Limitado número de estudos incluídos. Não inclusão de estudos desenvolvidos na região norte do país. Exclusão de artigos que analisavam o acesso a saúde da população trans juntamente com outros membros LGBTQIA+.	Compreender as políticas públicas atuais e criar novas que protejam os direitos dos indivíduos trans. Criar um modelo de atenção à saúde que integre sabores e mitigue os preconceitos no serviço de saúde. Criar formas de reduzir o estigma existente sobre as pessoas trans e problemas psicológicos.
9	Identificar fatores do processo de hormonização de pessoas transmasculinas a partir da ideia de agenciamentos e de cuidado.	Desrespeito e desconhecimento dos profissionais da saúde. Limitado número de serviços especializados. Patologização da transexualidade.	A análise etnográfica baseia-se apenas no acompanhamento de duas pessoas transmasculinas. É impossível generalizar os resultados do estudo.	Informar as múltiplas formas de existir de pessoas trans e transmasculinas. Rever a automedicação a partir de um olhar de agenciamento e cuidado. Repensar a formação de médicos, buscando qualifica-los para atuar no processo transexualizador.
